# Design and Implementation of Intelligent Agent Training Systems for Virtual Vehicles

**DOI:** 10.3390/s21020492

**Published:** 2021-01-12

**Authors:** Claudio Urrea, Felipe Garrido, John Kern

**Affiliations:** Department of Electrical Engineering, Universidad de Santiago de Chile, Av. Ecuador 3519, Estación Central, Santiago 9170124, Chile; felipe.garridos@usach.cl (F.G.); john.kern@usach.cl (J.K.)

**Keywords:** machine learning, intelligent agents, autonomous vehicle, reinforcement learning, behavioural cloning

## Abstract

This paper presents the results of the design, simulation, and implementation of a virtual vehicle. Such a process employs the Unity videogame platform and its Machine Learning-Agents library. The virtual vehicle is implemented in Unity considering mechanisms that represent accurately the dynamics of a real automobile, such as motor torque curve, suspension system, differential, and anti-roll bar, among others. Intelligent agents are designed and implemented to drive the virtual automobile, and they are trained using imitation or reinforcement. In the former method, learning by imitation, a human expert interacts with an intelligent agent through a control interface that simulates a real vehicle; in this way, the human expert receives motion signals and has stereoscopic vision, among other capabilities. In learning by reinforcement, a reward function that stimulates the intelligent agent to exert a soft control over the virtual automobile is designed. In the training stage, the intelligent agents are introduced into a scenario that simulates a four-lane highway. In the test stage, instead, they are located in unknown roads created based on random spline curves. Finally, graphs of the telemetric variables are presented, which are obtained from the automobile dynamics when the vehicle is controlled by the intelligent agents and their human counterpart, both in the training and the test track.

## 1. Introduction

In 1954, Farley et al. simulated a neural network on a computer, adding the notions of weights and thresholds [1]. In 1955 McCarthy et al. organized a conference on Artificial Intelligence (AI) in which they delivered a presentation on artificial neural network [2]. In 1958, Rosenbalt published an article that introduced the concept of perceptron, which took the eye’s retina as a reference [3]. Since then, the advances reached through these theories, as well as the power of the computer calculations available nowadays, allow for conducting research and simulation oriented to the development of autonomous vehicles in the field of artificial intelligence.

A work conducted by Pfeiffer et al. [4] focuses on planning the movement of autonomous robots. The study deals with an algorithm for an individual robot to learn how to navigate towards a target based on expert demonstrations. The learned navigation model is transferrable not only to virtual environments but also to real, and even unknown, settings. While imitating, the robot learns how to avoid collisions. The main disadvantage mentioned in this study is the poor performance of the algorithm in open spaces with reflective surfaces as training was conducted using ideal conditions. The authors propose that perhaps retraining with real data could reduce this defect. The robot used was a Kuboki Turtle Bot that employed information from the environment –supplied by a laser range finder sensor– and used convolutional neural networks for the planning of the trajectory to be followed.

Chen et al. [5] presents a study in which information is extracted from images to plan the movement of an autonomous automobile.

From the emergence of the first videogames, programmers and scientists have committed themselves to create AI algorithms for computers to imitate and compete with their human counterpart. For example, there are multiple chess, backgammon and go engines, as well as RPG, MOBA, shooter games, which have defeated world champions. This has given rise to the so-called e-sports [6,7,8]. In addition, some companies in this industry have released platforms for the development of videogames, for example, Cryengine, Unreal Engine and Unity [9]. 

Currently, most videogame developers are assisted by IAs in the design and evaluation process. To use them, two characteristics are assessed: Skills and game style, in order to quantify “how human” an IA is. This type of application is different from the common use given to IAs, which normally compete to defeat human players. Nevertheless, testing their training, satisfaction and/or acceptance degree before commercializing a videogame is challenging [10,11,12]. 

Regarding autonomous vehicles, most part of the current applications are based on a set of semantic definitions related to the tasks these vehicles need to learn and perform in motion. Additionally, tests to measure performance quantitatively during these tasks are implemented. These applications integrate field tests and simulated tests—which are correlated—and whose assessment is conducted through quantitative performance indexes for each predefined task [13,14,15]. 

Most methods and applications associated with autonomous vehicles include sensors of different nature. This has made deep multi-modal learning popular. These autonomous vehicles should anticipate or predict the movement of the detected objects, –such as people or animals–, which is fundamental to avoid accidents during their trajectory. In this context, some object detection techniques such as Faster R-CNN, One Stage Detector (OSD), Two Stage Detector (TSD) and Single Shot Detector (SSD) are usually utilized [16,17,18,19]. 

## 2. State of Research on Intelligent Agents

The concept “Intelligent Agent” (IA) has been defined by several authors, most of whom agree on the fact that an IA is an entity, computer system or software that perceives its surroundings by means of sensors, and that executes actions to achieve the goals set by a designer. Some of its characteristics are autonomy, learning capacity, reasoning, reactive behavior, mobility social skills, and perception of its environment and goal-oriented behavior. Besides the characteristics above, an IA is considered to exhibit other human traits such as knowledge, intention, and emotions [9,20,21].

In 2017, Shah et al. [22] created a simulator called Airsim for testing and developing AI algorithms in Unreal Engine, which offered realistic physical and graphical representations. This simulator yielded good results when a flying quadrotor was simulated both in the software and in the real world.

In the field of Machine Learning (ML), an approach widely used for IA learning is Reinforcement Learning (RL), which originates from behavioral psychology and neuroscience [23]. RL provides a framework for IAs to learn in the same way humans do, i.e., through sequential interaction with their environment and other agents. These IAs develop decision-making sequences that maximize the reward for a future goal. Table 1 presents a summary of RL types based on IAs, which are used in the modeling [24].

On their part, Bennewitz et al. [25] present an approximation that considers movements and ML. The authors propose a learning technique based on the collection of trajectories that characterize typical movement patterns of people. The information to be used, which originates from the environment, is gathered by a rangefinder laser and grouped using the expectation maximization algorithm. This generates a Markov model for estimating the current and future position of people, through which, for example, a mobile robot equipped with a laser beam and a camera can predict the position of elements in its surroundings. This approximation can be applied to the improvement of mobile robot navigation. In these cases, a laser and a camera are used, which are two elements frequently employed in videogames. This laser generates a beam denominated Raycast (Raycast: Function that releases a beam with arbitrary direction and origin and returns –among other variables– the distance from the object that is intercepting.).

Machine learning has been used in the creation of autonomous vehicles. Since conducting these experiments with real automobiles could be expensive and/or dangerous, simulators like CARLA (https://carla.org/) have been developed, which allow for the validation and training of autonomous driving systems [26,27,28,29,30,31,32,33,34,35,36,37]. Other simulators like TORCS (https://sourceforge.net/projects/torcs/) have been used with entertainment purposes, but also as AI research platforms [38]. In this way, ML could be applied to the driving of a motorized vehicle within a three-dimensional (3D) environment [39].

For a long time, software agents have been considered a key technology for diverse control applications due to their operation based on distributed architectures. From this departure point, different works have been conducted. 

In Ref. [40], a negotiation-based control approach is proposed to deal with the variability within a production system, where the agents involved can operate with direct communication and interaction.

A multiagent architecture based on the cloud and autoreactive is presented in [21]. This architecture allows the agents, clients and production entities subscribed to exchange information in real time.

In Ref. [41], a large number of Multi Agent System (MAS) patterns is compared, which leads to the conclusion that such agents could tremendously benefit the design of Cyber-Physical-Production-Systems (CPPS). In the same work, MAS-based production is discussed as an efficient approach for addressing the complexity of CPPS development.

In Ref. [9], Unity has been used as a simulation environment for ML algorithms due to its graphic capacities, physical and programming representations, and its “ML-Agents” plug-in, which was especially created with this purpose.

In the field of manufacturing system representation, several studies have been conducted using modeling languages. In this context, the automatic initialization of each agent has been proposed as a pre-created knowledge basis based on semantic web technologies [42]. This approach allows for verifications of specification coherence and a more efficient communication centered on the aspects that require real time management. 

In Ref. [43], manufacturing systems based on scalable and flexible agents are presented, whose production plan is created by autonomous agents that exploit a semantic description of web-based artifacts.

In another vein, in Ref. [44] an ontology-based model is proposed for the abstraction of devices and components as a way of allowing their interaction and achieving a plug and produce function.

## 3. Problem to Be Addressed

If there autonomous vehicles existed, passengers could be transported from A to B without human intervention. For years, aircraft navigation systems have had automatic pilot devices and can fly thousands of miles on their own, albeit always supervised by humans through a pre-set course. However, to date, the implementation of semi-automation in motorized terrestrial vehicles, like automobiles, has been very complex. Additionally, experiments on real vehicles, whose mass and potential energy are high, are not an easy task either, making the process costly and risky. Nevertheless, thanks to the advent of 3D engines and physics solvers, a virtual vehicle with a behavior similar to that of its real counterpart was created. Therefore, if the simulation of a virtual vehicle with an AI system are adequately combined, a vehicle could be controlled autonomously in the real world.

In this paper, the design, simulation, training, and implementation of a virtual automobile programmed in C# language with its own codes is presented. This brings the advantage of having access to simulation data and, in turn, being able to command the physical parameters of the vehicle. The techniques explored in this study are Imitation Learning (IL) and RL.

IL is a method that feeds from expert demonstrations [45,46]. Therefore, in this paper, the design and implementation of a movement platform with 2 Degrees of Freedom (DoF), which allows for emulating the main controls of a vehicle, are also presented. In this way, the human expert has a steering wheel, stereoscopic vision, and pedals, among other elements. Conversely, RL requires the adequate tailoring of a reward function, which encourages the IA to make the best decisions in order to obtain the best possible gain [46].

An IA can be trained using a data set from which general rules can be created. After training, the IA will be able to deal with similar problems successfully. Therefore, following these guidelines, this paper models a close loop lane with traffic barriers using the Blender software, in order to provide a training track. Conversely, for the trial stage, which uses spline curves, a script is implemented that generates a road with a transversal section identical to the test track, but with a random trajectory.

## 4. Material and Methods

The design and implementation of intelligent agent training systems for virtual vehicles addressed in this work could be classified as a physics-based motion study in which the position, speed, and acceleration of a virtual vehicle are governed by Newton’s laws. A virtual vehicle is implemented considering mechanisms that represent accurately the dynamics of a real automobile, such as motor torque curve, suspension system, differential, and anti-roll bar, among others. A control interface for the automobile movement is designed and implemented by building a 2-DoF physical platform. This interface is used by a human expert in the case of the IA trained by imitation. The platform is equipped with a virtual reality system, movement cancelling and control commands, among other elements. To drive this automobile, IAs are designed and implemented, and then trained by IL and RL, which also enables the comparison of different driving methods. 

To achieve this, the following steps are presented below:

1. An application with high-quality graphic capacities, which is also able to provide physical representations realistic and suitable for the simulation of a real automobile, is created. In this way, an IA could be trained and tested when taking over such an automobile.
2. A movement platform with control commands and Virtual Reality (VR) adequately linked to the same was designed to act as a human control interface. This platform provides the user with stereoscopic vision, a steering wheel, pedals, and movement signs.
3. Both training and test tracks are developed.
4. Once the IAs are trained, their performances are compared to that of the human expert on both tracks.

### 4.1. Analysis and Selection of Learning Platforms

An analysis of the main learning platforms currently being researched in the AI field is presented below:

1. Arcade Learning Environment (ALE) [47]: A simple framework oriented to objects that allows researchers to develop IA for Atari 2600 games. However, it lacks realistic physical representations, and its graphs are over-simplistic.
2. DeepMind Lab [48]: A 3D game platform in the first person, designed for AI and ML system researchers. It can be used to study how autonomous artificial agents learn complex tasks in larger realms, partly observed and visually diverse. DeepMind Lab uses the graphic engine of the videogame Quake III and lacks realistic physical representations.
3. Project Malmo [49]: A platform designed for research and experiments on AI. It is based on the videogame Minecraft, has polygonal graphs and poor graphic representations.
4. Unity [9]: Videogame engine that has a Graphic User Interface (GUI). It provides rendering of high-quality graphs (close to photo realistic), contains physical representations of Nvidia PhysX (Nvidia PhysX is a proprietary mid-layer software engine and development kit designed to perform very complex physical calculations. PhysX is a proprietary “middleware” software layer engine and development kit designed to carry out very complex physical calculations. https://www.ecured.cu/PhysX) and supports scripts in C# programming language. In relation to its simulation potential, the calculations conducted by Unity are independent from rendered photograms and the simulation parameters can be changed during use. Additionally, this software has a free version that offers most of the features in the paid version.
5. UE4: High quality videogame engine with realistic graphics. It has a plug-in for TensorFlow. However, both user documentation and quantity of demos offered by the program are poor compared to Unity. Therefore, after the analysis of the main learning platforms currently in use on AI research, Unity was selected for the design and implementation of the development environment presented in this paper, due to its outstanding features.

### 4.2. Advantages of the Proposed Research

The main advantages of the proposed research are presented in Table 2.

## 5. Design of the Virtual Vehicle

**Figure 1:**
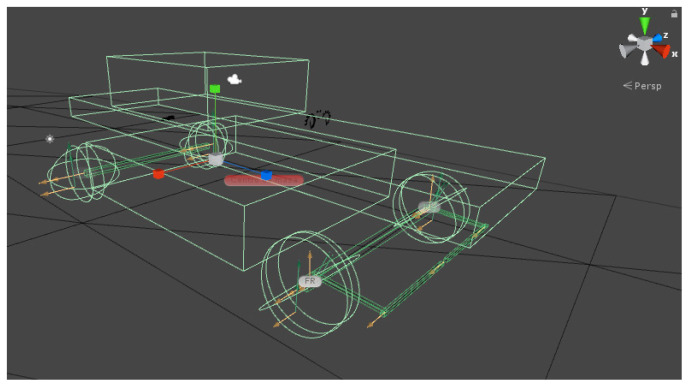
Elements of the virtual automobile.

**Figure 2:**
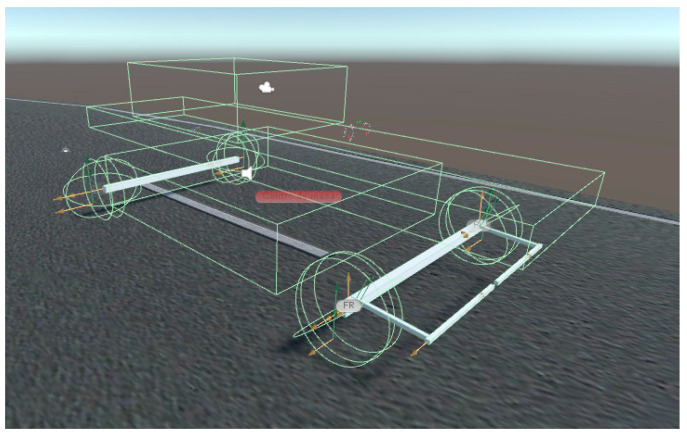
Elements of the virtual automobile (image rendered in Unity).

**Figure 3:**
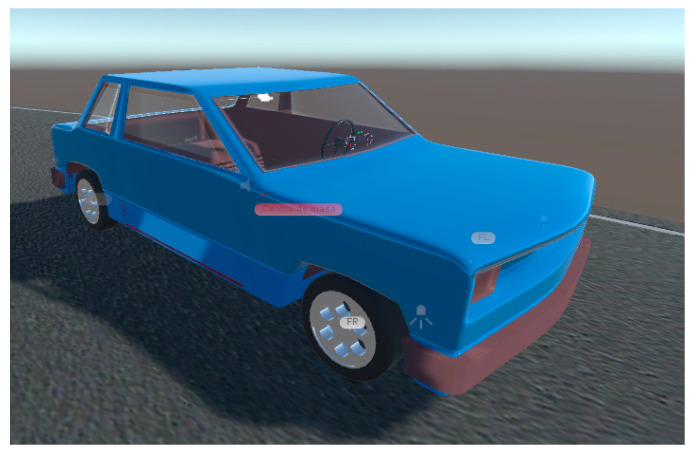
Virtual automobile with avatar.

The virtual vehicle requires to represent the dynamic behavior or a real automobile accurately. Table 3 shows the design characteristics considered [50]:

From these considerations, the virtual vehicle is designed using the following objects and physical components offered by Unity presented below in Table 4:

Figure 1 and Figure 2 show the virtual vehicle designed, with the joints marked by yellow arrows and the colliders structures in green. Figure 3 presents the vehicle with an overlapped rendered avatar, which was modeled using Blender. Additionally, a path that has an asphalt texture is created. The scene presents shadows caused by the sunlight and a skybox that simulates a clear sky. The avatar allows for visualizing more realistically the virtual automobile on the road, but without interacting with the dynamic characteristics of the vehicle.

### 5.1. Control Interface

**Figure 4:**
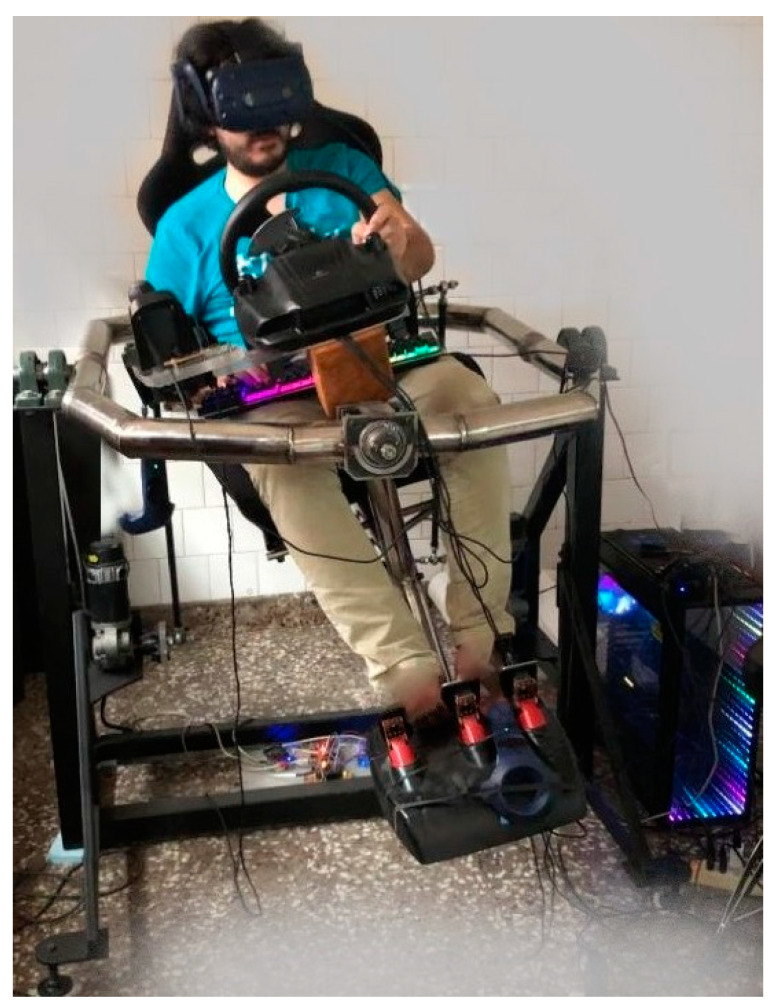
Grid system of IA (physical platform).

The control interface of the vehicle’s movement corresponds to a physical platform equipped with 2-DoF, which comprises of the following components shown in Table 5:

Figure 4 shows the control interface for the movement of the vehicle implemented. It may be observed how this physical movement platform is being employed in the training of IA. Movement cancelling is achieved via software, using the rotation of one of the hand controllers coupled to the movement physical platform.

### 5.2. Training Track

**Figure 5:**
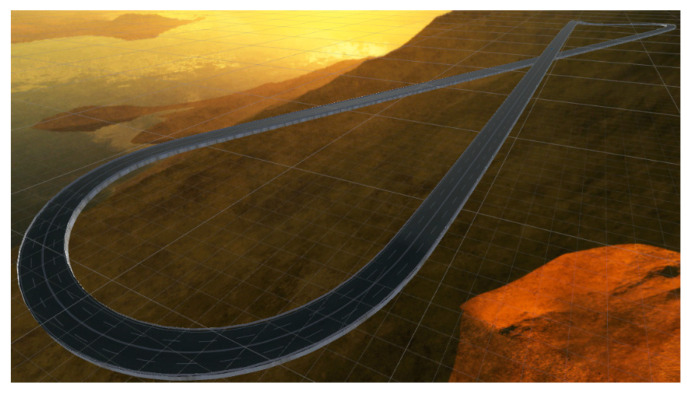
Training track.

The IAs are trained in the track shown in Figure 5. This track has been designed with an “8” shape and its length is 2000 m, has four lanes with a width of 15 m in total, a slope whose difference in height is 20.1 m, and backstops.

### 5.3. Test Track

**Figure 6:**
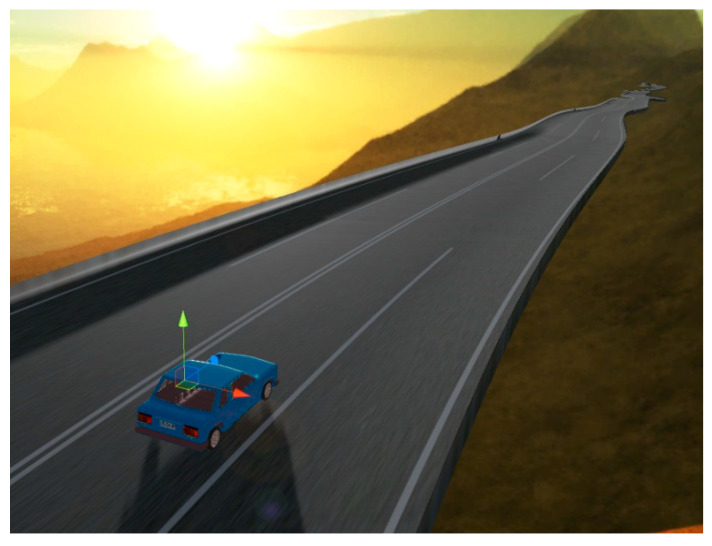
Road generated with cubic splines and random control points.

The test tracks are designed using a script that generates a soft trajectory from the sections of cubic spline curves. Then, a two-dimension profile is extruded through these trajectories, thereby creating a road. The splines designed with random control points give the script enough flexibility to create multiple geometries in the highways. In particular, this research work considers 64 sections of splines with control points that allow for generating tracks where the IAs are tested. Figure 6 shows that the road is not uniform and that has backstops.

## 6. Theory/Calculation

The two experiments conducted for the IA to learn are an algorithm for learning by reinforcement ‒called Proximal Policies Optimization (PPO)‒ and learning by imitation, using the Behavioral Cloning (BC) algorithm [51,52].

The IA has two action vectors: Torque and steering, and perceives its environment through one, or a combination of the following techniques: Distance toward obstacles (roadside barriers) by means of raycasting, speed, and acceleration.

Evidently, the IA has not access to the road map, and the rewards or penalties in the case of RL would be dependent on at least the following elements: Avoiding high *g* (9.81 m/s^2^) forces, avoiding collisions, and not being detained.

### 6.1. Implementation of Learning by Reinforcement and Imitation

RL has three fundamental elements: observation of the environment, the IA’s actions, and the reward or prize received by the IA. However, IL methods do not involve any reward or prize. The next subsection deals with how the RL and IL for the vehicle are designed and implemented.

#### 6.1.1. Observation Vectors

It must be noted that in this study, IAs do not have any type of artificial vision of their environment. Nevertheless, the variables that the IA senses for its environment are constituted by 23 continuous vectors [-1, 1]. These observation vectors are: 10 remote field Raycast, 10 close field Raycast, normal speed, tangential speed, and acceleration.

**Figure 7:**
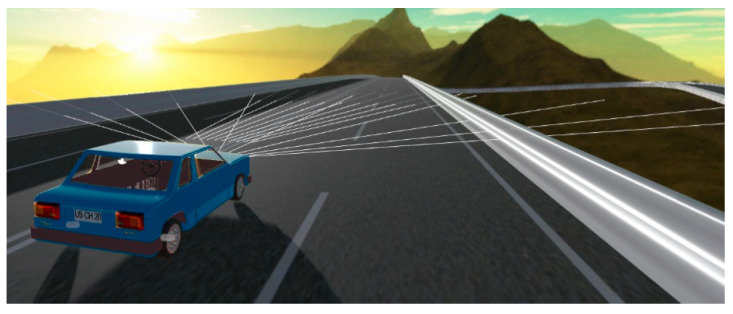
Virtual automobile deploying its distance detection rays on the training track.

Figure 7 shows the virtual vehicle on the training track. The sensing between the vehicle and its remote and close surroundings can be observed thanks to the distance detector rays the virtual vehicle projects. 

According to the classification presented in Li et al. [18], IAs could be assessed based on two criteria: Scenario-based testing and functionality-based testing. The first one judges the degree of intelligence thanks to which the IA reaches or not specific objectives, for example, whether an IA achieves circulation without any collision, or detecting a traffic sign. The second criterion establishes that a completely intelligent vehicle is one that executes all the functions that a human expert usually performs, such as visually recognizing vehicles, signs, pedestrians, or animals. Therefore, considering this classification, the IAs trained in this research would be closer to being evaluated according to their functionality, which is understood as the capacity of controlling the speed and direction of the virtual automobile in a way similar to a human expert. However, as presented by Li et al. [13], the assessment of the performance of the trained IAs should also be encompassed by a subjective appraisal, for example, a human could perfectly evaluate the quality of an IA in driving a car by simply observing the movement, maneuvers, and telemetric statistics that the IA generates. 

#### 6.1.2. Reward Function

The mathematical-logical functions that assess the performance of IAs trained by reinforcement to generate new behaviors, reinforcing or punishing the previous actions, are designed based on the criteria shown in Table 6:

Mathematically speaking, each of these criteria are implemented by means of the following functions,

(1) $$R_{distance}=-\sum_{r=1}^{20}\frac{0.5}{d_{r}}$$

(2) $$R_{speed}=\left\{ \begin{array}{ccc} -0.1\cdot f & if & v<0\\ 2\cdot f & if & v>0\\ v\cdot f & if & 2<v<12\\ 0.1\cdot f & if & 12<v<27 \end{array}\right.$$

(3) $$R_{collision}=-0.1\cdot f$$

(4) $$R_{acceleration}=-0.1\cdot f\cdot a$$

(5) $$R_{steeringangle}=-0.01\cdot steering^{2}$$

where *R* represents the reward; the sub-indexes represent distance, speed, collision, acceleration, and steering angles, respectively; *d_r_* represents the distance perceived by each detector ray in meters; *v* represents the tangential speed of the virtual automobile, in m/s; *a* represents acceleration, in m/s^2^; *steering angle* is the rotational angle, in degrees, reached by the steering wheel and the parameter *f* represents a dimensionless factor that allows for adjusting gains. In the experiment, a factor *f* = 0.1 is selected so the award function is defined for each step as:

(6) $$R=R_{\mathrm{distance}}+R_{speed}+R_{acceleration}+R_{\mathrm{steering}\;\mathrm{angle}}$$

Based on these criteria, the virtual vehicle can circulate at a safe distance, respecting the road barriers; moving at a constant and positive speed as long as possible; avoiding collisions and driving in a straight line most of the time. The following algorithm shows the programming of these criteria.
**Algorithm 1** Award accumulated in each IA’s observation-action cyclefor i = 1:20 if ray[i].hit = true Add reward(−0.5/ray[i].distance); end end if velocity ≤ 0 AddReward(−0.1 *· f*); if velocity > 0 AddReward(2 *· f*); if 2 < velocity < 12 AddReward(velocity *· f*); if 12 < velocity ≤ 27 AddReward(0.1 *· f*); AddReward(−0.1 · acceleration); AddReward(−0.01 · steer · steer);

#### 6.1.3. Action Vectors

Action vectors define how an IA reacts to stimuli, and they are designed based on the following considerations: Steering, where positive angles imply a turn to the right and negative angles imply a turn to the left; and torque, where positive torque means acceleration and negative torque means brake.

The PPO and BC training methods are configured using numerical sets called hyperparameters. These values include both the characteristics of the neural networks to be trained and the behavior of the algorithm during its convergence process. Below, the definition of the hyperparameters used are presented in Table 7:

ML-Agents gives the user the possibility of building these hyperparameters, which are structured in two dedicated text files based on the IA training method (PPO or BC). In the training phase, the user can modify these hyperparameters depending on IA behavior. In this paper, for PPO and BC, the hyperparameters specified in Table 8 and Table 9 are employed.

To assess the performance of both IA after training, the data generated during the virtual automobile’s movement are recorded as a comparative tool. By means of these data, both the control signals and the dynamic performance of the vehicle are numerically identified. The next section specifies the telemetric data collected.

## 7. Results

This section presents the comparison of the telemetric results of the variables representing the real vehicle when this circulates on both the training and the test tracks. For each execution, the data set to be compared is registered on a list. To this end, a script is designed, which allows for exporting all these data in a simple text file. By means of a calculation table, the information to be delivered can be processed at the same time as a set of graphs that show the evolution of the automobile variables:

- *Gas*: A positive torque –representing how open or closed is the flow of fuel to the motor– is obtained for positive values, which enables the vehicle to speed up. Instead, negative values imply that the automobile activates its brakes.
- *Steering*: This angle allows for controlling the automobile’s steering wheel. Positive values translate into turns to the right, while negative ones indicate turns to the left.
- *Lateral acceleration*: This acceleration is the result of variation in the direction angle.
- *Longitudinal acceleration*: This acceleration is the result of gas variation.
- *RPM*: Number of turns the motor crankshaft conducts per minute.
- *Instant rapidity*: The speed vector tangential to the automobile trajectory.
- *Gear*: Number of gears used by the automobile transmission. The vehicle considered has five gears in total.

Regarding the training time of IAs, this varies greatly depending on the method used. In the case of the PPO algorithm, the training time is 10 h approximately, processing 100,000 steps at a speed 10 to 20 times the real time. Conversely, the BC method takes only some minutes to imitate the human expert. All the physical calculations of the simulations are renderized in a fixed 5 ms interval.

### 7.1. Case 1: Telemetry on the Training Track

**Figure 8:**
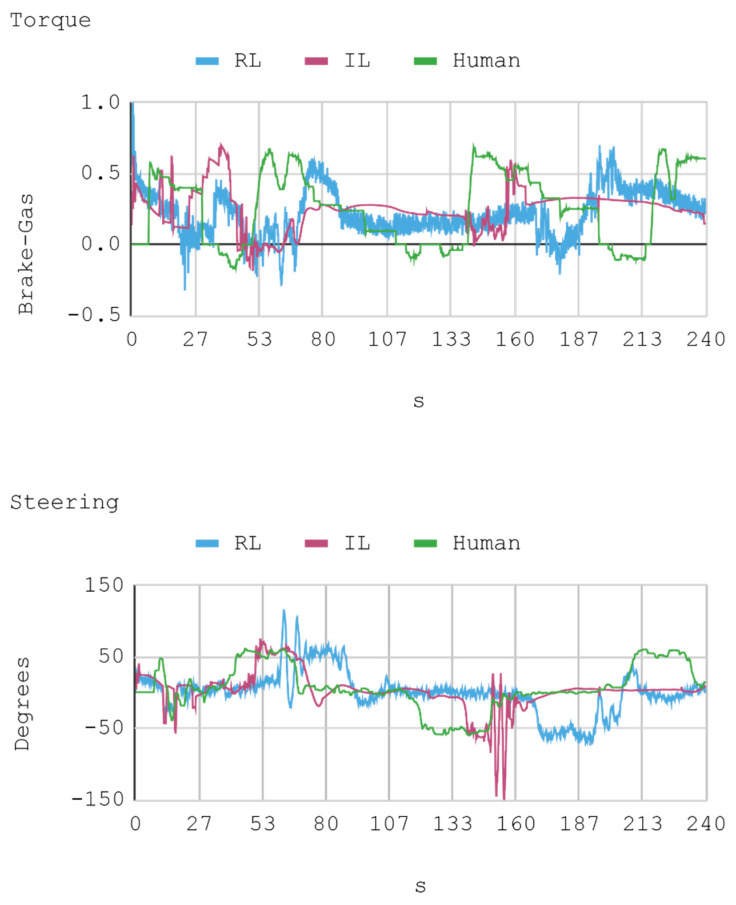
Case 1. Automobile torque and steering.

**Figure 9:**
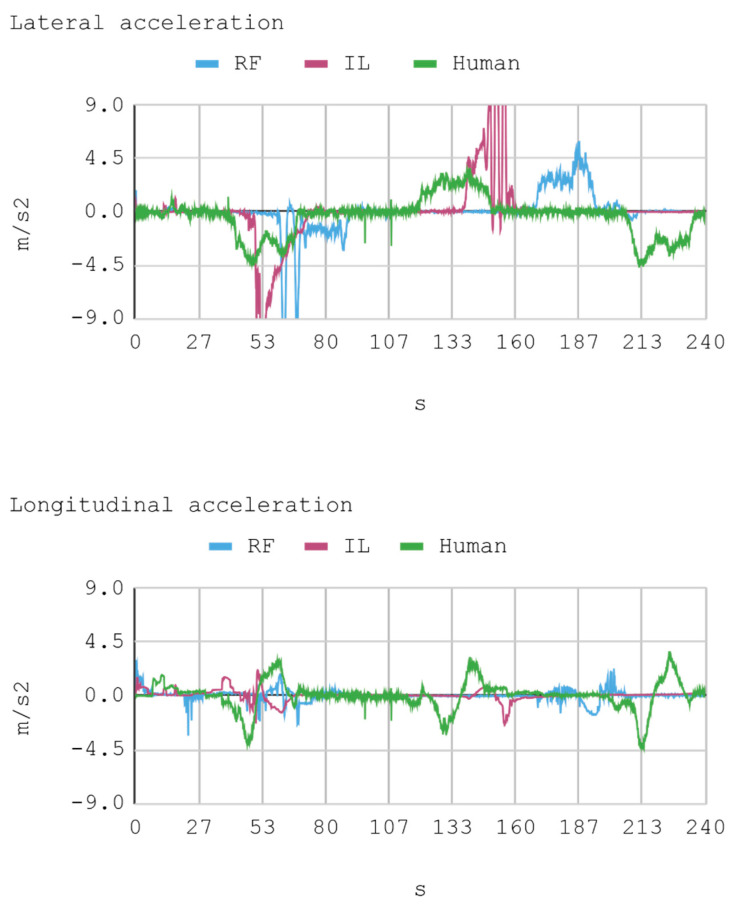
Case 1. Lateral and longitudinal acceleration of the automobile.

**Figure 10:**
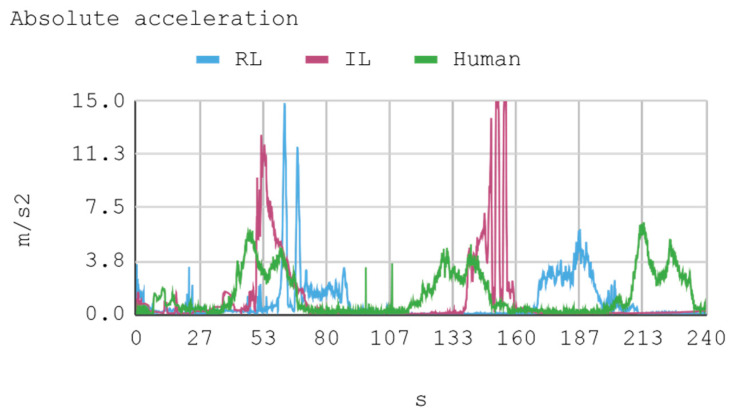

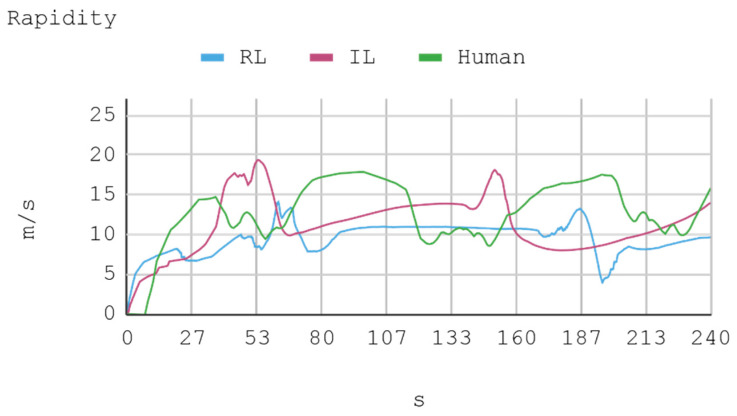
Case 1. Automobile’s absolute acceleration and rapidity.

**Figure 11:**
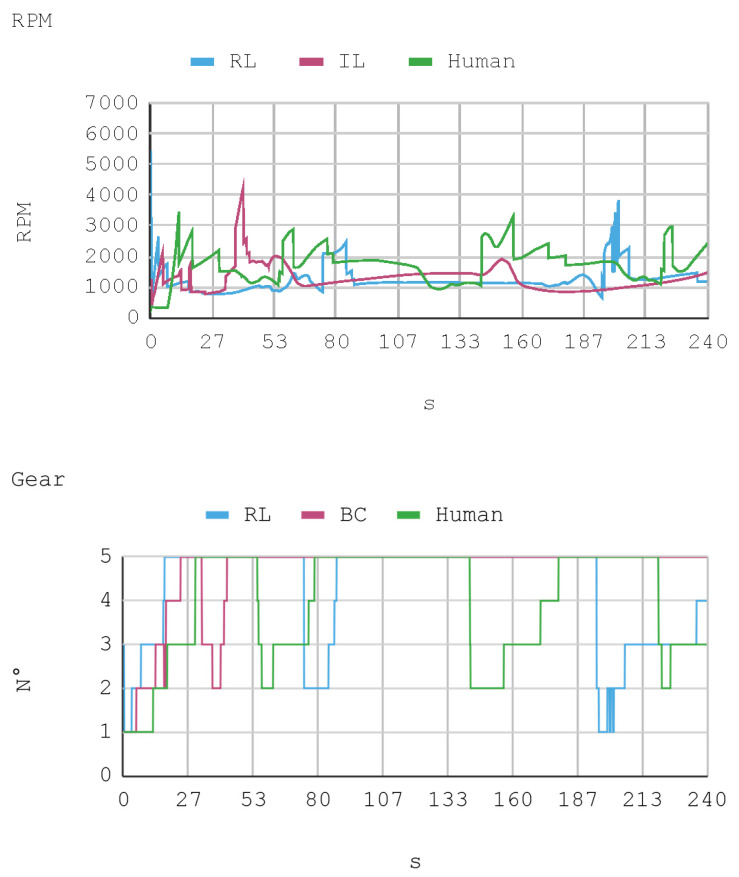
Case 1. Automobile’s revolutions per minute and gear number.

Telemetric data from both IAs (RL and IL), as well as the human expert, driving the automobile on the training track were obtained for this case. These data are presented by means of graphs, considering a time interval of 240 s as it is the approximate time for an automobile to complete, at least, one lap in a test track. Each sample is captured with a sampling frequency of 10 Hz. The first experimental results show the presence of jitter in the torque and direction controls of the IA trained by reinforcement. To soften this behavior, a moving average filter is designed and employed, thereby improving the results for direction control but not for torque control. Therefore, the graphs below consider a filter in the direction control for the IA trained by reinforcement. In Figure 8, Figure 9, Figure 10 and Figure 11, the metric estimators of the automobile can be observed. In the training of IA by RL a behavior with noise both in the torque and direction controls indicates that the automobile suffers small vibrations during its trajectory, which is reflected clearly in its net acceleration. Conversely, training IA by BC, the torque and direction controls exhibit a more refined behavior similar to that of a human expert.

**Figure 12:**
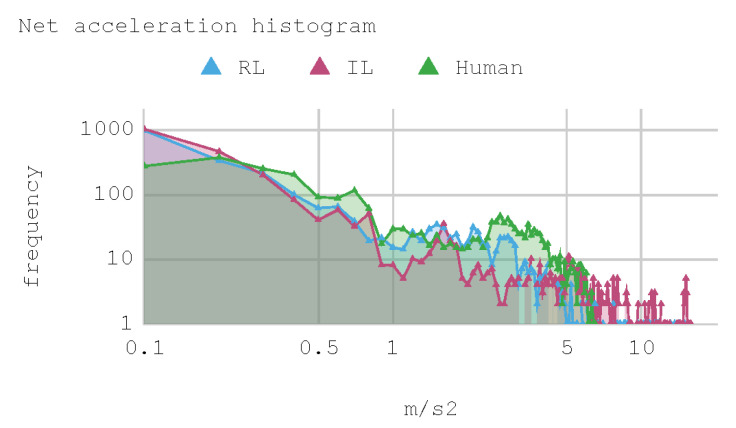
Case 1. Net acceleration histogram for training track.

Figure 12 presents a histogram of the net acceleration distribution during the 240 s of test, grouped in 200 intervals with a size equal to 0.1 m/s^2^. This histogram allows for observing the frequency of all 2400 samples captured for both the IAs and the human expert. In addition, the IAs display sporadic accelerations that surpass 7 m/s^2^ more than once. Table 10 presents a summary of Case 1.

The RPM and gear values that complement the other dynamic variables of the vehicle show the consistency of the results. This is because, for example, when an automobile requires higher torque (in positive slopes), gears are lowered so RPMs increase. However, in straight trajectories (without positive slopes), there is a tendency to use the fifth gear.

### 7.2. Case 2: Telemetry on the Test Track

**Figure 13:**
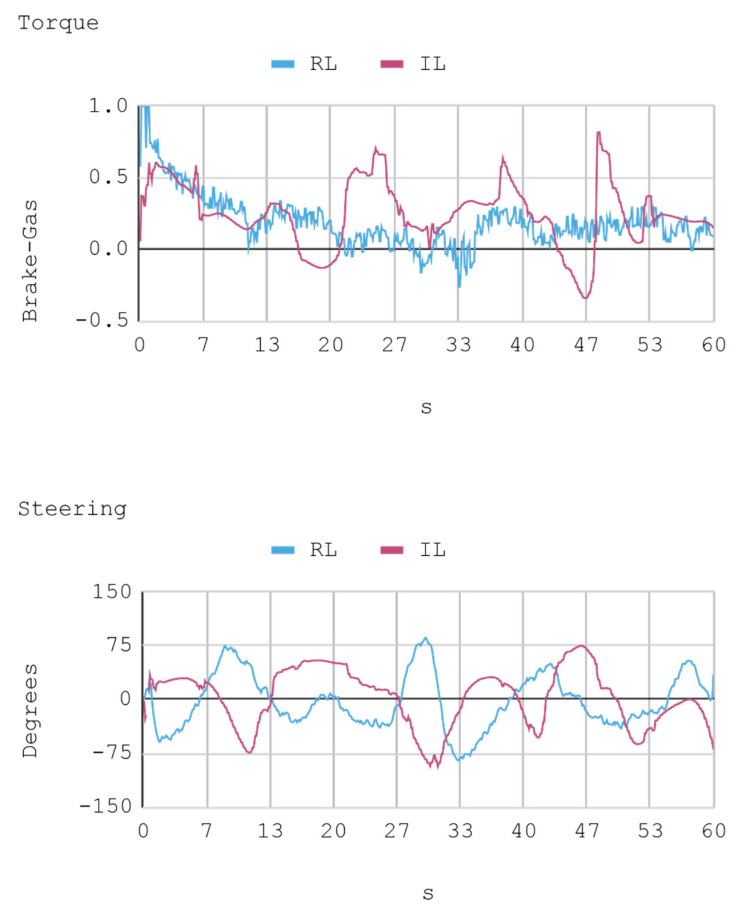
Case 2. Automobile’s torque and steering.

**Figure 14:**
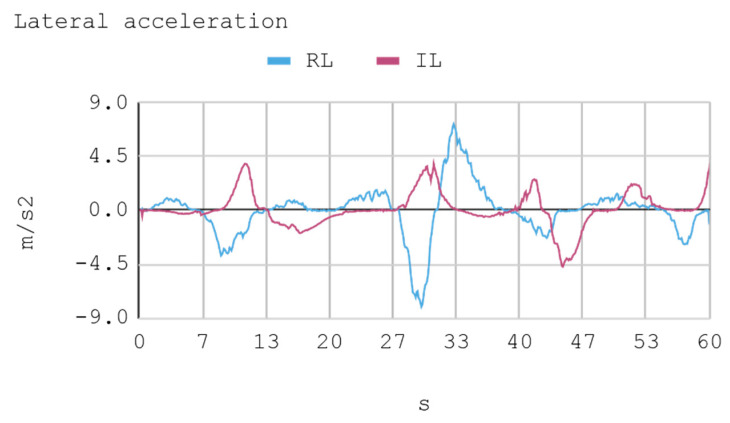

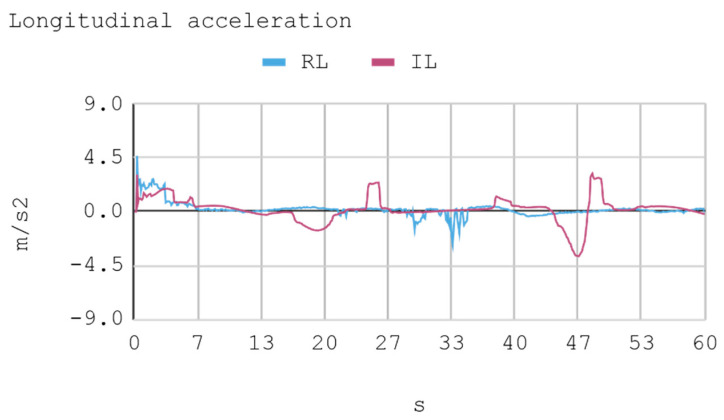
Case 2. Lateral and longitudinal acceleration of the automobile.

**Figure 15:**
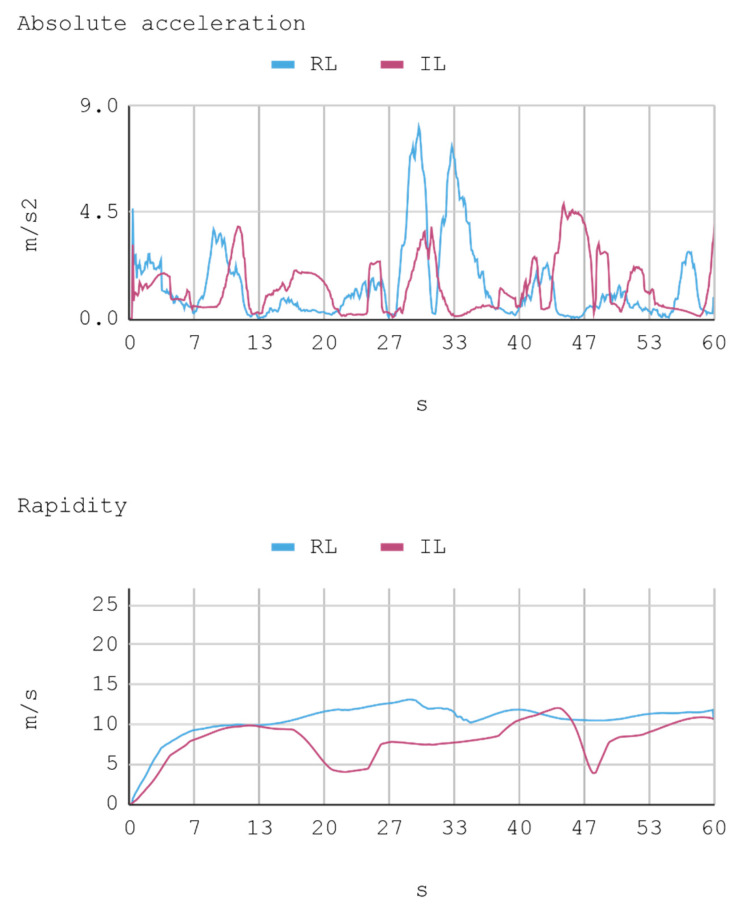
Case 2. Automobile’s absolute acceleration and rapidity.

**Figure 16:**
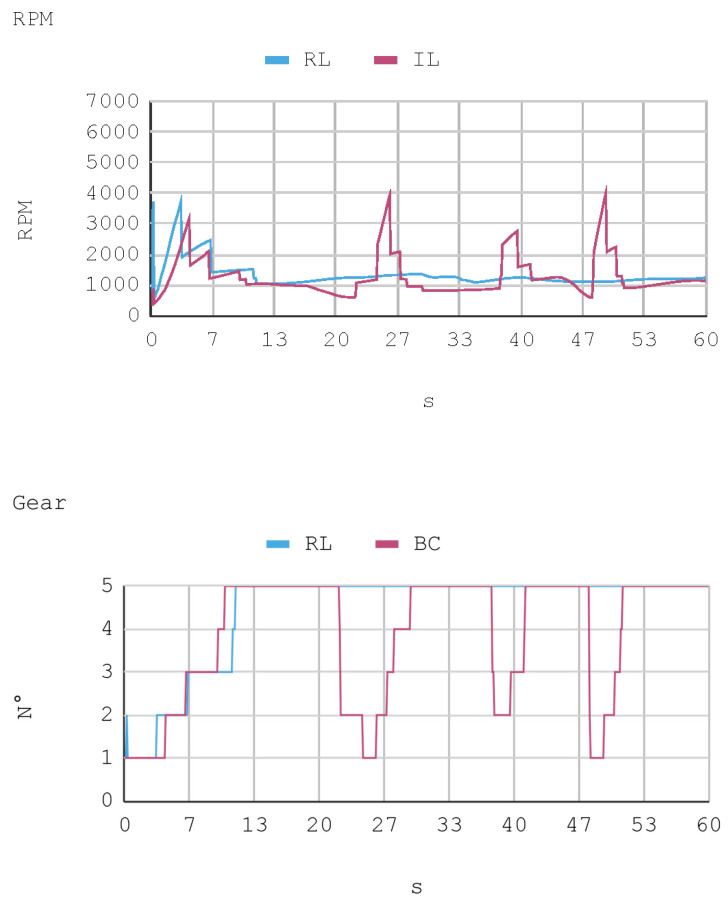
Case 2. Automobile’s revolutions per minute and gear number.

In this case, telemetric data for both IA (RL and IL) were employed when they were driving the automobile on the test tracks. It should be noted that the test tracks are not the same for RL and IL, since these have been created based on the parameters that enable some randomness in their morphologies, in order to represent in the most realistic way the infinite possibilities of vehicle track designs. Nevertheless, obviously, a similar level of morphological difficulty is maintained between the tracks to be used for both IAs. The telemetric data considered are presented through graphs, taking a time interval of 60 s, although for statistical calculations 240 s are taken into account. Each sample is captured with a sample frequency of 10 Hz. Figure 13, Figure 14, Figure 15 and Figure 16 present the metric estimators of the vehicle. Table 11 presents a summary of Case 2. 

## 8. Discussion

### 8.1. Case 1: Telemetry on the Training Track

In general, the human counterpart achieves a more precise global control of the automobile. This outcome is attributed to the fact that the human expert has several practice minutes before developing the skill, which may be seen in the graphs obtained. It must be noted that its net acceleration in the moments of highest stress oscillates between 2 m/s^2^ and 6 m/s^2^. In fact, in the routes “curve to the right ascending and then descending” (at second 50 and 234, respectively), and “curve to the left ascending and then descending” (at second 120), net accelerations present values within that range.

The IA trained by imitation loses both direction and torque control more often than the IA trained by reinforcement. This occurs with both track curves at seconds 53 and 140, approximately. Nevertheless, in the straight routes on the tracks no significant differences are appreciated since both the AIs and the human expert have an adequate command of the automobile.

As for instant rapidity, the IA trained by reinforcement has a smooth performance close to the speed of maximum reward (around 12 m/s) when driving in straight segments. Conversely, the IA trained by imitation tends to be more reckless, reaching speeds higher but less stable than those exhibited by the IA trained by reinforcement. On his part, the human expert achieves a more average speed, in such a way that in 240 s he makes 1.25 laps in the circuit approximately. In the training of IA by RL, on average, an absolute acceleration lower than in the training of IA by BC is observed, with an effective acceleration value of 0.55 m/s^2^ and 0.84 m/s^2^, respectively. However, the human expert achieves 0.63 m/s^2^ in that same circuit.

### 8.2. Case 2: Telemetry on Test Tracks

During IA training by RL, a lower absolute acceleration may be seen on average than in the training by BC, with an effective acceleration value of 0.79 m/s^2^ and 0.89 m/s^2^, respectively. Regarding mean rapidity, the IA trained by BC reaches 10.44 m/s, while the IA trained by RL achieves 10.84 m/s.

**Figure 17:**
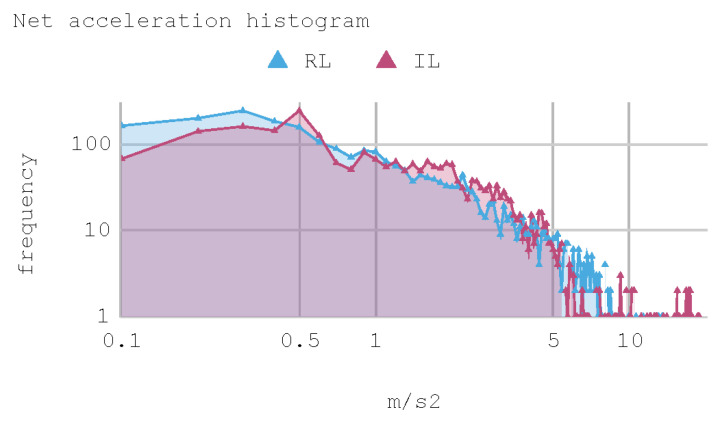
Case 2. Histogram of net acceleration on test tracks.

Figure 17 contains a histogram with the distribution of net acceleration during the 240 s of tests, grouped in 200 intervals with a size of 0.1 m/s. These histograms show the occurrence frequency of each of the 2400 samples captured for the two IAs. Again, more than once, the IAs present sporadic accelerations that exceed 7 m/s^2^ along the tests.

From a functional point of view, it can be asserted that both IAs are components and have enough AI to control the virtual vehicle both in terms of speed and direction in the straight segments. In the zones with curves and slopes, although the IAs survive and manage to complete both tracks (training and test) without colliding, the quality of the control exerted decreases to the extent of presenting a behavior far from the control executed by a human expert. Additionally, considering the difficulty-based criterion on the scenario that simulates a four-lane highway and specifically assessing direction, torque, and quantity of collisions, the IAs are competent for all these requirements separately.

## 9. Conclusions and Future Work

This work presented the design, simulation and implementation of a virtual automobile equipped with mechanisms that represent real components, such as motor torque curve, suspension system, differential, and anti-roll bar. To achieve this, the video platform Unity and the ML-Agents library were used. A control interface for the automobile movement was designed and implemented by building a 2-DoF physical platform. This interface was used by a human expert in the case of the IA trained by imitation. The platform was equipped with a virtual reality system, movement cancelling and control commands, among other elements. To drive this automobile, first IAs were designed and implemented, and then trained by IL and RL, which also enabled the comparison of different driving methods. A training environment for the IA was built in Unity with capacity to control the automobile by taking 23 input data and performing two actions as output. Regarding the IAs performance on the training track, it is concluded that the IA trained by reinforcement presents the lowest effective acceleration, surpassing even the human expert. In the case of the IA trained by imitation, this exhibits a performance below its counterpart trained by reinforcement but surpasses it in mean speed by 18%. This is due to the reward function implemented, which compels the IA trained by reinforcement to seek those policies that ensure its subsistence in the highway, avoiding high *g* forces and collisions, among other elements. However, in contrast, this IA has a mean speed lower than the three IAs actors considered. Occasionally, both IAs generate transient accelerations of large amplitude and short duration, which even reach values above 1 *g*. The movement of a human being in a vehicle with these accelerations would not be comfortable. However, the human expert that controls the virtual automobile through the movement simulator did not generate these transient accelerations. During the test phase, both IAs demonstrated being competent in the control of the automobile, circulating without difficulties on an unknown track for more than 4 min. On the other hand, net effective acceleration increased by almost 6% for the IA trained by imitation, and by 43% for the IA trained by reinforcement. Additionally, both IAs presented an increase in abrupt accelerations, although from a practical perspective, this would not imply a disadvantage necessarily because, for example, in an unmanned or an exploration vehicle such accelerations are discarded. Therefore, it is concluded that IAs were successfully trained and, thus, they can control a virtual automobile. 

In this research, microcontroller programming codes were used to control the robotized platform implemented, scripts for handling and generating 3D objects, serial communication without latency and VR technologies were also designed integrated and simulated. The computer simulations for the automobile were conducted with a very realistic physical representation thanks to an adequate analysis and synthesis of basic components such as curve toque of an Otto cycle motor, suspension system and anti-roll bar. 

In relation to the review of current research on autonomous vehicles and its associated algorithms, it can be asserted that an advantage of the method proposed in this work is that IAs are effectively trained through a simulation that includes sensors similar to those of real vehicles. In addition, while the virtual vehicle was stopped, it was started by the IAs as a consequence of the environment stimuli. In addition, being able to accelerate the simulation time and of visualizing the progress during the training stage allow for refining the configuration of the hyperparameters and the coefficients of the reward function.

Using a human control interface like the one considered in this work opens the possibility to study algorithms for generating reward functions based on expert demonstrations.

Given the flexibility offered by intelligent agent training systems for virtual vehicles developed in this work, any future study or implementation in such systems could be tested from both the AI and a functional perspective, comparing the results in terms of the performance of human experts. Therefore, the results in this work can be extended to several contexts related to machine learning. However, as specific future research, it is planned to adapt the IAs designed to interact with a real industrial or mobile robot.

An explanatory video on the design and implementation of intelligent agent training systems for virtual vehicles developed in this work can be found in the Supplementary Materials.

## Figures and Tables

**Table 1:** Summary of RL types based on IAs, which are used in the modeling [24].

Reinforcement Learning Type	Description	Reference
Bush-Mosteller (BM)	A type of statistical learning where a predictive function is derived from data.	Bush & Mosteller (1955) ^1^
Learning Automata (LA)	Simple algorithm operating in a stochastic environment where agents can improve their actions during operation.	Narendra & Thathachar (1974) ^2^
Q-Learning (QL)	A policy, expressed as a matrix of values for states and actions, is learned so an agent knows what to do in different circumstances. It does not require a model of the problem to be solved. (State→Action→Reward).	Watkins (1989) ^3^
State → Action → Reward → State → Action (SARSA)	Extends Q-Learning by also considering the future selected state-action. Uses a model it builds.	Rummery & Niranjan (1994) ^4^
Temporal-Difference (TD)	Learning from experience without an environmental model, which updates estimates before outcome is known.	Sutton (1988) ^5^

^1^ (Bush, R.R.; Mosteller, F. Stochastic Models for Learning. *John Wiley & Sons, Oxford, UK*, **1955**); ^2^ (Narendra, K.S.; Thathachar, M.A. Learning Automata—A Survey. *IEEE Transactions on Systems, Man, and Cybernetics*, **1954**, 4, 323–334); ^3^ (Watkins, C.J. Learning from Delayed Rewards. *PhD Thesis, King’s College, London, UK,* **1989**); ^4^ (Rummery, G.A.; Niranjan, M. On-Line Q-Learning Using Connectionist Systems, Technical Report CUED/F-INFENG/TR 166. *University of Cambridge, Department of Engineering, Cambridge, UK*, **1994**); ^5^ (Sutton, R.S. Learning to Predict by the Methods of Temporal Differences. *Machine Learning*, **1988**, *3*, 9–44.).

**Table 2:** Advantages of the proposed research.

Features
Development of open programming codes.
Promotion of the use of free software, such as: Unity3d and its Unity ML-Agents Toolkit.
Supports virtual reality.
The user can train IAs using the driving simulator.
Democratizes Access to ML research.
The system proposed, as an open system, would support the incorporation of new algorithms, the creation of training environments for other types of robots and/or learning paradigms.

**Table 3:** Vehicle components and their design features.

Component	Design Characteristics
Traction and suspension	Front-wheel drive. Suspension with a rigid axle in the front and the rear. Anti-roll bar in the front axle. Ackerman steering geometry and differential.
Weight	1000-kg mass.
Engine	100 HP maximum power at 5500 rpm. 134 Nm maximum torque at 3500 rpm (includes look up table with torque curve).
Transmission	Automatic 5 speed transmission.
Controls	Acceleration pedal. Brake pedal. Steering wheel.

**Table 4:** Objects and physical components.

Unity Objects	Components
Rigid bodies	Mass.
Joints	Hinge and ball joints.
Colliders	Box and capsule colliders.

**Table 5:** Simulator components.

Components	Detail
Actuators	Two direct current electric motors. 12 V; 1350 W peak, and 28:1 gear box equipped with two optical quadrature encoders –one for each motor– that estimate the turning angle of the motors. Two Pololu VNH519 H bridges of 12 V and 30 A, respectively, model. Driven from a 12 V and 60 A power source.
Microcontrollers	Two Arduino Uno development boards; the first one estimates the rotation angle of both motors by means of interruptions via hardware and the second one to achieve the serial communication and control of the two H bridges by PWM signals.
Steering wheel and pedals	Logitech g27 steering wheel.
Virtual Reality System	HTC VIVE Pro Virtual Reality System. Headset and two hand controllers.

**Table 6:** Design criteria.

Feature	Detail
Distance of the virtual automobile from the road barriers	Punishment for the virtual automobile performance when this is too close to the road barriers.
Virtual automobile speed	Reward if speed is positive. Reward if speed is within a specific interval. Punishment if speed is negative.
Virtual automobile acceleration	Punishment for positive and negative acceleration.
Collisions	Punishment if the automobile collides with the road barriers.
Steering angles	Punishment if the virtual automobile is not at the left of the track.

**Table 7:** Hyperparameters and their descriptions.

Hyperparameter	Description
Batch size	Number of experiences within each iteration of the gradient descent. This number is expected to be a fraction of the buffer size.
Beta	Force with which entropy stabilizes. Entropy causes random policies through which the IA explores the action space during training.
Buffer size	Number of experiences gathered before updating the random policy model that allows for the learning of the IA.
Epsilon	Acceptable divergence threshold –while the gradient descent is being updated– between new and old random policies. This threshold allows for determining how fast the random policy model evolves during IA training.
Gamma	Discount factor for future rewards. This factor allows for determining whether the agent should value the current gains or worry about potential future rewards.
Hidden units	Number of units in the hidden layer of the neural network.
Lambda	Factor used to calculate the General Advantage Estimator (GAE). This factor allows for determining –after calculating a new value estimation– to what extent the IA is dependent on its previous value estimation compared to the new estimation. The IA could trust more in the previous estimation, or more in the rewards received from its environment (corresponding to the new value estimation).
Learning rate	Initial learning rate from the gradient descent. During the initial learning of the IA, this rate represents the weight of each update stage.
Max steps	Number of steps in the IA training process.
Num epoch	Number of iterations, through the experience buffer, when conducting the gradient descent optimization.
Num layers	Number of hidden layers in the neural network.
Time horizon	Number of experience steps gathered before sending them to the experience buffer.

**Table 8:** Configuration hyperparameters for the IA trained by reinforcement through the PPO algorithm.

Trainer	PPO
Batch size	16
Beta	0.01
Buffer size	256
Epsilon	0.15
Gamma	0.9
Hidden units	64
Lambda	0.9
Learning rate	5 × 10^−4^
Max steps	10 × 10^4^
Num epoch	10
Num layers	3
Time horizon	4

**Table 9:** Configuration hyperparameters for the IA trained by reinforcement through the BC algorithm.

Trainer	BC (Online) ^1^
Batch size	16
Hidden units	128
Learning rate	5 × 10^−5^
Max steps	5 × 10^5^
Batches per epoch	2
Num layers	5
Time horizon	2

^1^ Online emphasizes that learning is conducted in real time, that is to say, the IA and the human expert circulate on the training track at the same time.

**Table 10:** Case 1. Mean speed and statistics derived from acceleration of IAs and human expert on the training track.

Statistics/Actor	IA IL	IA RL	Human
Mean speed (m/s)	11.22	9.75	12.8
Effective acceleration (m/s^2^)	0.84	0.55	0.63
Abs(a) mode, bin = 0.1 (m/s^2^)	0.1	0.1	0.2
Abs(a) statistical frequency	1.012	970	369
Abs(a) median (m/s^2^)	0.12	0.16	0.52
Abs(a) standard deviation (m/s^2^)	2.44	1.53	1.51
Abs(a) maximum (m/s^2^)	15.7	14.9	6.5
N over 6 (m/s^2^)	123	35	13

**Table 11:** Case 2. Mean speed and statistics derived from net acceleration of IAs on the test track.

Statistics/Actor	IA IL	IA RL
Mean speed (m/s)	10.44	10.84
Effective acceleration (m/s^2^)	0.84	0.79
Abs(a) mode, bin = 0.1 (m/s^2^)	0.5	0.3
Abs(a) statistical frequency	246	247
Abs(a) median (m/s^2^)	1.09	0.77
Abs(a) standard deviation (m/s^2^)	2.22	2.06
Abs(a) maximum (m/s^2^)	18.7	56.4 ^1^
N over 6 (m/s^2^)	66	74

^1^ This high value originates from some glitch in the physics of Unity. No collisions were observed during the test stage.

## Data Availability

Not applicable.

## Supplementary Materials

The following are available online at https://www.mdpi.com/1424-8220/21/2/492/s1, Video S1: Explanatory video on the design and implementation of intelligent agent training systems for virtual vehicles.

## Abbreviations

**Initials**	**Meaning**
ALE	Arcade Learning Environment
AI	Artificial Intelligence
BC	Behavioral Cloning
BM	Bush-Mosteller
CPPS	Cyber-Physical-Production-System
DoF	Degrees of Freedom
GAE	General Advantage Estimator
GUI	Graphic User Interface
IA	Intelligent Agent
IL	Imitation Learning
QL	Q-Learning
LA	Learning Automata
ML	Machine Learning
MAS	Multi Agent System
OSD	One Stage Detector
PPO	Proximal Policy Optimization
RL	Reinforcement Learning
SSD	Single Shot Detector
SARSA	State-Action-Reward-State-Action
TD	Temporal-Difference
TORCS	The Open Racing Car Simulator
TSD	Two Stage Detector
VR	Virtual Reality

## References

[1] Farley B., Clark W. (1954). Simulation of self-organizing systems by digital computer. Trans. IRE Prof. Group Inf. Theory.

[2] McCarthy J., Minsky M.L., Rochester N., Shannon C.E. (2006). A proposal for the dartmouth summer research project on artificial intelligence, August 31, 1955. AI Mag..

[3] Rosenblatt F. (1958). The perceptron: A probabilistic model for information storage and organization in the brain. Psychol. Rev..

[4] Pfeiffer M., Schaeuble M., Nieto J., Siegwart R., Cadena C. From perception to decision: A data-driven approach to end-to-end motion planning for autonomous ground robots. Proceedings of the 2017 IEEE International Conference on Robotics and Automation (ICRA).

[5] Chen C., Seff A., Kornhauser A., Xiao J. DeepDriving: Learning affordance for direct perception in autonomous driving. Proceedings of the IEEE International Conference on Computer Vision (ICCV).

[6] Urbaniak K., Wątróbski J., Sałabun W. (2020). Identification of players ranking in e-sport. Appl. Sci..

[7] Risi S., Preuss M. (2020). From chess and atari to starcraft and beyond: How game ai is driving the world of ai. KI-Künstl. Intell..

[8] Tang X., Song H., Wang W., Yang Y. (2020). Vehicle spatial distribution and 3D trajectory extraction algorithm in a cross-camera traffic scene. Sensors.

[9] Juliani A., Berges V.P., Vckay E., Gao Y., Henry H., Mattar M., Lange D. (2018). Unity: A general platform for intelligent agents. Comput. Sci. Math..

[10] Kwon O. (2020). Very simple statistical evidence that alphago has exceeded human limits in playing go game. Comput. Sci..

[11] Urrea C., Kern J., Alvarado J. (2020). Design and evaluation of a new fuzzy control algorithm applied to a manipulator robot. Appl. Sci..

[12] Zhao Y., Borovikov I., Silva F.M., Beirami A., Rupert J., Somers C., Harder J., Kolen J., Pinto J., Pourabolghasem R. (2020). Winning isn’t everything: Enhancing game development with intelligent agents. IEEE Trans. Games.

[13] Li L., Wang X., Wang K., Lin Y., Xin J., Chen L., Xu L., Tian B., Ai Y., Wang J. (2019). Parallel testing of vehicle intelligence via virtual-real interaction. Sci. Robot..

[14] Gao H., Shi G., Xie G., Cheng B. (2018). Car-following method based on inverse reinforcement learning for autonomous vehicle decision-making. Int. J. Adv. Robot. Syst..

[15] Gao H., Shi G., Wang K., Xie G., Liu Y. (2019). Research on decision-making of autonomous vehicle following based on reinforcement learning method. Ind. Robot. Int. J..

[16] Lefèbre S., Vásquez D., Laugier C. (2014). A survey on motion prediction and risk assessment for intelligent vehicles. ROBOMECH J..

[17] Gao H., Cheng B., Wang J., Li K., Zhao J., Li D. (2018). Object classification using CNN-based fusion of vision and LIDAR in autonomous vehicle environment. IEEE Trans. Ind. Inform..

[18] Li L., Huang W.-L., Liu Y., Zheng N., Wang F.-Y. (2016). Intelligence testing for autonomous vehicles: A new approach. IEEE Trans. Intell. Veh..

[19] Feng D., Haase-Schutz C., Rosenbaum L., Hertlein H., Glaser C., Timm F., Wiesbeck W., Dietmayer K. (2020). Deep multi-modal object detection and semantic segmentation for autonomous driving: Datasets, methods, and challenges. IEEE Trans. Intell. Transp. Syst..

[20] Wooldridge M., Jennings N.R. (1995). Intelligent agents: Theory and practice. Knowl. Eng. Rev..

[21] Mishra N., Singh A., Kumari S., Govindan K., Ali S.I. (2016). Cloud-based multi-agent architecture for effective planning and scheduling of distributed manufacturing. Int. J. Prod. Res..

[22] Shah S., Dey D., Lovett C., Kapoor A. (2018). AirSim: High-fidelity visual and physical simulation for autonomous vehicles. Distributed Auton. Robotic Syst..

[23] Sutton R., Barto A. (1998). Reinforcement learning: An introduction. IEEE Trans. Neural Netw..

[24] Brearcliffe D., Crooks A. (2020). Creating Intelligent Agents: Combining Agent-Based Modeling with Machine Learning. https://easychair.org/publications/preprint/w3H1.

[25] Bennewitz M., Burgard W., Cielniak G., Thrun S. (2005). Learning motion patterns of people for compliant robot motion. Int. J. Robot. Res..

[26] Pouliquen M., Bernard A., Marsot J., Chodorge L. (2007). Virtual hands and virtual reality multimodal platform to design safer industrial systems. Comput. Ind..

[27] Dosovitskiy A., Ros G., Codevilla F., Lopez A., Koltun V. CARLA: An open urban driving simulator. Proceedings of the 1st Annual Conference on Robot Learning.

[28] Urrea C., Venegas D. (2018). Design and development of control systems for an aircraft. Comparison of performances through computational simulations. IEEE Lat. Am. Trans..

[29] Li L., Ota K., Dong M. (2018). Humanlike driving: Empirical decision-making system for autonomous vehicles. IEEE Trans. Veh. Technol..

[30] Pérez L., Diez E., Usamentiaga R., García D.F. (2019). Industrial robot control and operator training using virtual reality interfaces. Comput. Ind..

[31] Urrea C., Saa D. (2020). Design and implementation of a graphic simulator for calculating the inverse kinematics of a redundant planar manipulator robot. Appl. Sci..

[32] De Bruyne J. (2020). Driving autonomous vehicles. Rev. Droit Technol. Inf..

[33] Minhas R.A., Javed A., Irtaza A., Mahmood M.T., Joo Y.-B. (2019). Shot classification of field sports videos using AlexNet convolutional neural network. Appl. Sci..

[34] Miclea R.-C., Dughir C., Alexa F., Sandru F., Silea I. (2020). Laser and LIDAR in A system for visibility distance estimation in fog conditions. Sensors.

[35] Urrea C., Matteoda R. (2020). Development of a virtual reality simulator for a strategy for coordinating cooperative manipulator robots using cloud computing. Robot. Auton. Syst..

[36] Gao H., Zhu J., Zhang T., Xie G., Kan Z., Hao Z., Liu K. (2020). Situational assessment for intelligent vehicles based on Stochastic model and Gaussian distributions in typical traffic scenarios. IEEE Trans. Syst. Man, Cybern. Syst..

[37] Palmerini L., Klenk J., Becker C., Chiari L. (2020). Accelerometer-based fall detection using machine learning: Training and testing on real-world falls. Sensors.

[38] Wymann B., Espié E., Guionneau C., Dimitrakakis C., Coulom R., Sumner A. (2000). Torcs, the Open Racing Car Simulator. http://torcs.sourceforge.net.

[39] Cha M., Yang J., Han S. (2008). An interactive data-driven driving simulator using motion blending. Comput. Ind..

[40] Mezgebe T.T., El Haouzi H.B., Demesure G., Pannequin R., Thomas A. (2020). Multi-agent systems negotiation to deal with dynamic scheduling in disturbed industrial context. J. Intell. Manuf..

[41] Salazar L.A.C., Ryashentseva D., Lüder A., Vogel-Heuser B. (2019). Cyber-physical production systems architecture based on multi-agent’s design pattern-comparison of selected approaches mapping four agent patterns. Int. J. Adv. Manuf. Technol..

[42] Ocker F., Kovalenko I., Barton K., Tilbury D.M., Vogel-Heuser B. (2019). A framework for automatic initialization of multi-agent production systems using semantic web technologies. IEEE Robot. Autom. Lett..

[43] Ciortea A., Mayer S., Michahelles F. Repurposing manufacturing lines on the fly with multi-agent systems for the web of things. Proceedings of the 17th International Conference on Autonomous Agents and MultiAgent Systems.

[44] Orio G.D., Rocha A., Ribeiro L., Barata J. The PRIME semantic language: Plug and produce in standard-based manufacturing production systems. Proceedings of the International Conference on Flexible Automation and Intelligent Manufacturing 2015 (FAIM’15).

[45] Hussein A., Gaber M.M., Elyan E., Jayne C. (2017). Imitation learning: A survey of learning methods. ACM Comput. Surv. (CSUR).

[46] Kaelbling L.P., Littman M.L., Moore A.W. (1996). Reinforcement learning: A survey. J. Artif. Intell. Res..

[47] Mnih V., Kavukcuoglu K., Silver D., Graves A., Antonoglou I., Wierstra D., Riedmiller M. Playing atari with deep reinforcement learning. Proceedings of the NIPS Deep Learning Workshop 2013.

[48] Beattie C., Leibo J.Z., Teplyashin D., Ward T., Wainwright M., Küttler H., Lefrancq A., Green S., Valdés V., Sadik A. (2016). Deepmind Lab. https://deepmind.com/research.

[49] Johnson M., Hofmann K., Hutton T., Bignell D. The Malmo Platform for Artificial Intelligence Experimentation. Proceedings of the 25th International Joint Conference on Artificial Intelligence (IJCAI).

[50] Rill G. (2009). Vehicle Dynamics.

[51] Schulman J., Wolski F., Dhariwal P., Radford A., Klimov O. (2017). Proximal Policy Optimization Algorithms. https://arxiv.org/pdf/1707.06347v2.pdf.

[52] Bain M., Sammut C. (1999). A framework for behavioural cloning. Mach. Intell..

